# The Role of Ultrasound in the Diagnosis and Treatment of Cellulite: A Systematic Review

**DOI:** 10.3390/jcm15030943

**Published:** 2026-01-23

**Authors:** Dora Intagliata, Maria Luisa Garo

**Affiliations:** 1 Poliambulatorio ID Future, 96100 Siracusa, Italy; 2 Biostatistics and Research Methodology Unit, Mathsly Research, 89900 Vibo Valentia, Italy

**Keywords:** cellulite, ultrasound imaging, diagnostic assessment, treatment monitoring, esthetic medicine

## Abstract

**Background**: Cellulite is a highly prevalent condition with dermal and subcutaneous alterations poorly captured by visual grading systems. Ultrasound has emerged as a non-invasive imaging modality capable of objectively quantifying morphological features relevant to cellulite. This systematic review evaluated the evidence on ultrasound for the diagnosis, structural characterization, and treatment monitoring of cellulite, identifying methodological limitations and research gaps. **Methods**: This systematic review (PROSPERO:CRD420251185486) followed the PRISMA statement. Searches were conducted in PubMed, Scopus, and CENTRAL up to November 2025. Risk of bias was evaluated using ROBINS-I and the Newcastle–Ottawa Scale. **Results**: Nine studies involving 785 participants were included. Ultrasound frequencies ranged from 12 to 35 MHz, with some scanners operating across broader bandwidths. Despite variability in devices, acquisition protocols, and clinical comparators, all studies consistently demonstrated that ultrasound quantifies key structural characteristics of cellulite. Diagnostic investigations reported moderate-to-strong correlations (r ≈ 0.31–0.64) between ultrasound-derived measures and clinical severity scores. Interventional studies showed measurable reductions in dermal and subcutaneous thickness, decreased adipose protrusion height, and improved dermal echogenicity across multiple treatment modalities. Ultrasound frequently detected microstructural remodeling not readily visible on clinical examination. **Conclusions**: Ultrasound is a valuable imaging modality for objectively characterizing cellulite and monitoring treatment-induced tissue remodeling. Standardized acquisition protocols, validated analytic criteria, and larger controlled studies are needed to support integration into routine dermatologic and esthetic practice. The quantitative and reproducible nature of ultrasound-derived parameters also provides a suitable foundation for future integration with data-driven and artificial intelligence–based image analysis frameworks.

## 1. Introduction

Cellulite, clinically defined as edematous fibrosclerotic panniculopathy, is characterized by dimpling and irregularities of the skin surface resembling an “orange peel.” It predominantly affects women, with prevalence estimates of 80–98% among postpubertal females, reflecting its substantial esthetic and psychological burden [1]. Beyond cosmetic implications, cellulite is associated with diminished self-esteem and reduced quality of life [2,3].

Its pathophysiology is multifactorial, involving microcirculatory alterations, adipocyte hypertrophy, hormonal influences, and structural remodeling of fibrous septa and adipose tissue architecture [1,4,5,6]. These processes lead to structural changes in the dermal and subcutaneous compartments and have informed the development of therapeutic strategies targeting microcirculatory dysfunction, septal tension, and localized adiposity [7,8,9,10]. Traditionally, cellulite assessment relies on subjective visual scales and photographic analyses, which are limited by diagnostic imprecision and considerable inter-observer variability [11,12,13]. Anthropometric indices such as body mass index (BMI) or limb circumference show weak correlations with cellulite severity, further underscoring the need for objective and reproducible diagnostic tools [14,15,16]. Advances in imaging have therefore driven increasing interest in techniques capable of objectively characterizing subcutaneous tissue architecture and providing quantitative, operator-independent parameters [11,17,18]. In this context, ultrasound has emerged as a non-invasive and accessible modality enabling real-time visualization of adipose tissue, fibrous septa, edema, and dermal alterations [3,4,19,20,21]. Ultrasound-derived parameters have shown meaningful correlations with cellulite severity, supporting its role as a more objective alternative to traditional visual grading systems [16,22]. Moreover, ultrasound facilitates the identification of morphological subtypes and complements established clinical scales such as the Nürnberger–Müller classification, enabling individualized treatment planning and reliable monitoring of therapeutic response over time [3,15,17,23]. Despite its potential and growing use in clinical research, the literature on the clinical application of ultrasound in cellulite remains heterogeneous.

The primary aim of this systematic review was to evaluate the role of ultrasound in the diagnosis and treatment monitoring of cellulite. Specifically, we sought to (1) summarize the diagnostic accuracy and reliability of ultrasound for grading and characterizing cellulite; (2) describe the contribution of quantitative ultrasound parameters to the assessment and monitoring of treatment effects; (3) examine the relationships between ultrasound findings and established clinical or photographic grading scales; and (4) identify methodological limitations and gaps in the evidence to inform future research and support the standardization of ultrasound use in esthetic and dermatologic practice.

## 2. Materials and Methods

This systematic review was conducted according to the PRISMA 2020 statement [24] and registered in the PROSPERO database (CRD420251185486). The PRISMA checklist is reported as Supplementary Materials.

The components of the PICO question were: (population) adult individuals (≥18 years old) with clinically diagnosed cellulite of any severity and anatomical location; (intervention) ultrasound-based assessment using any modality, including B-mode, high-frequency ultrasound, Doppler, or elastography, applied for diagnostic evaluation, morphological characterization, severity grading, or treatment monitoring; (comparator) clinical grading scales, photographic assessment, alternative imaging techniques such as MRI, or anthropometric measures when available; (outcome) diagnostic performance measures, quantitative ultrasound parameters or inter- and intra-observer agreement.

### 2.1. Inclusion and Exclusion Criteria

We included studies enrolling adults (≥18 years) with clinically diagnosed cellulite, irrespective of anatomical location or severity grade. Eligible studies assessed ultrasound-based techniques for diagnostic evaluation, characterization of tissue structure, grading of cellulite severity, or monitoring of treatment response. Studies in which ultrasound was used exclusively to measure changes following a therapeutic intervention were included only when the ultrasound parameters were relevant to the structural characterization of cellulite (e.g., dermal thickness, subcutaneous architecture, adipose protrusions). Studies evaluating only circumferential or surface-level cosmetic outcomes without structural ultrasound assessment were excluded. Both randomized and non-randomized designs were eligible, including randomized controlled trials, cohort studies, case–control studies, and cross-sectional studies. Exclusion criteria encompassed studies involving minors, animal models, or dermatologic conditions unrelated to cellulite. Case reports, editorials, narrative reviews, and conference abstracts were also excluded.

### 2.2. Information Sources

The following electronic databases were searched without language or date restrictions: PubMed (last searched: 14 November 2025), Scopus (last searched: 14 November 2025), and CENTRAL—Cochrane Central Register of Controlled Trials (last searched: 14 November 2025). Additional sources included backward and forward citation searching of all included studies, manual screening of reference lists, and consultation of study registries for potentially relevant trials. Only published peer-reviewed studies were considered.

### 2.3. Search Strategy

A systematic search for relevant studies was conducted in all cited databases from September to November 2025 without time or language restrictions using the following search strategy: (cellulite OR panniculopathy OR (edematous AND fibrosclerotic AND panniculopathy) OR gynoid lipodystrophy) AND (ultrasound OR ultrasonography OR high-frequency ultrasound OR B-mode OR Doppler OR elastography). No filters or limits were applied other than the exclusion of non-human studies.

### 2.4. Selection Process

The full list of articles retrieved from the systematic search was uploaded to Rayyan (https://www.rayyan.ai, accessed on 15 November 2025) and screened independently by two reviewers. After removal of duplicates, titles and abstracts were assessed using predefined eligibility criteria. Potentially relevant studies were subsequently evaluated in full text by the same two independent reviewers. Any discrepancies arising during either the title/abstract screening or full-text assessment were resolved through discussion, and consultation with a third external reviewer was planned if consensus could not be reached. No automation tools beyond Rayyan’s organizational interface were used.

### 2.5. Data Collection Process

Data extraction was performed independently by the two reviewers using standardized and piloted extraction forms to ensure consistency and accuracy. Prior to full extraction, the reviewers completed a calibration exercise on a subset of studies to harmonize data interpretation and reduce discrepancies. Extracted information included study characteristics (authors, year, country, design, sample size), participant demographics (age, sex, eligibility criteria), cellulite severity, and detailed descriptions of the ultrasound methodology (device type, probe frequency, imaging settings, acquisition protocol, and measurement procedures). Outcomes collected encompassed diagnostic performance measures, types of quantitative ultrasound parameters such as dermal and subcutaneous thickness, echogenicity patterns, configuration of fibrous septa, and presence of edema, as well as inter- and intra-observer reliability metrics. No study authors were contacted for additional information because all included studies reported sufficient methodological and outcome information to enable qualitative synthesis. Any discrepancies between reviewers were resolved through discussion until consensus was reached.

### 2.6. Outcomes and Additional Data

The following outcomes were sought from each study: (1) Diagnostic performance of ultrasound; (2) Type of quantitative ultrasound parameters (dermal thickness, subcutaneous fat thickness, echogenicity, septa visualization, tissue homogeneity); (3) Reproducibility outcomes (inter- and intra-observer agreement). All available results compatible with these domains were extracted, regardless of measurement scale, time point, or analytic approach.

### 2.7. Risk of Bias Assessment

The risk of bias was assessed independently by two reviewers using Newcastle–Ottawa Scale (NOS) for observational studies and ROBIN-I for interventistic studies. Any disagreements were resolved by consensus. No automation tools were used.

### 2.8. Synthesis Methods

The included studies were presented in chronological order to illustrate the historical progression of ultrasound applications in cellulite assessment and treatment monitoring. Subsequently, key findings were synthesized across diagnostic and treatment-monitoring domains to provide an integrated overview of the evidence. Since this review was conducted as a qualitative systematic synthesis without performing a meta-analysis, no statistical harmonization or quantitative pooling was required. Findings were therefore integrated through a structured narrative approach, supported by detailed tables describing study characteristics, ultrasound parameters, and treatment-related tissue changes. Heterogeneity across studies was examined qualitatively. Particular attention was given to variations in ultrasound methodology, cellulite severity, participant characteristics, and methodological rigor.

## 3. Results

### 3.1. Study Selection

**Figure 1:**
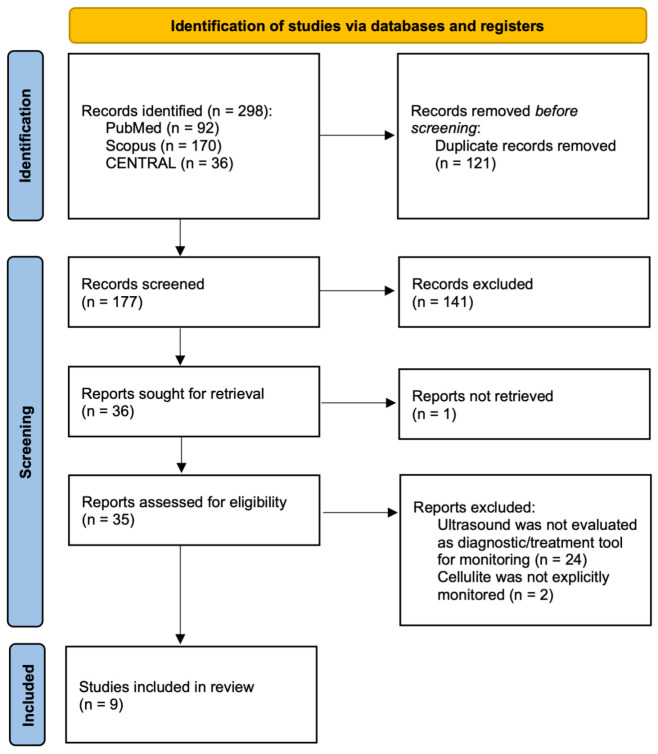
PRISMA Flowchart—Source: [24]. Study exclusion was performed without the use of automated systems.

A total of 298 records were identified through database searching, including 92 from PubMed, 170 from Scopus, and 36 from CENTRAL; no additional reports were identified through gray literature and citation chains. After removal of 121 duplicate records, 177 unique records underwent title and abstract screening. Of these, 141 records were excluded as they did not meet the inclusion criteria. A total of 36 full-text reports were sought for retrieval, of which one could not be obtained. The remaining 35 full-text articles were assessed for eligibility. Following full-text evaluation, 26 reports were excluded, primarily because ultrasound was not evaluated as a diagnostic or monitoring tool (*n* = 24) or because cellulite was not explicitly investigated (*n* = 2). Ultimately, nine studies met all predefined inclusion criteria and were included in the qualitative synthesis. The complete study selection workflow is illustrated in Figure 1.

### 3.2. Study Characteristics

The nine studies included in this review were conducted between 2008 and 2025 (Table 1). Early investigations focused primarily on the ability of ultrasound to delineate dermal–subcutaneous interface irregularities, while subsequent studies expanded the application of ultrasound to the monitoring of tissue changes following cosmetic or pharmacological interventions. More recent contributions further refined the use of ultrasound by integrating elastography, Doppler assessment, and more sophisticated morphological classifications.

Sample sizes ranged from 26 participants [13] to 150 [17]. All studies enrolled adult women with clinically confirmed cellulite, typically located on the buttocks, posterior thighs, or lateral thighs. Four studies evaluated the effects of topical agents [18,26,28], mechanical treatments or injectable therapies [29]. Five studies used observational or diagnostic designs [13,15,17,25,27], with the most recent developing a structured ultrasound-based classification for stage III cellulite.

Across all studies, high-frequency ultrasound was used to quantify structural skin and subcutaneous alterations associated with cellulite severity or treatment response. Frequencies ranged from 12 MHz to 35 MHz, with several investigations employing 20 MHz HFUS systems or 18 MHz linear radiologic probes. Three studies incorporated 3D ultrasound or elastography to enhance structural characterization. Standardization of the acquisition process varied across studies, although only two investigations provided detailed methodological controls.

Clinical comparators were similarly heterogeneous. The Nürnberger–Müller scale was used in several studies, whereas photonumeric clinical grading scales were adopted in two studies. Additional comparators included thigh circumference measurements, bioimpedance analysis, dermatologist-rated photographic scales, and elasticity assessments.

### 3.3. Risk of Bias

**Figure 2:**
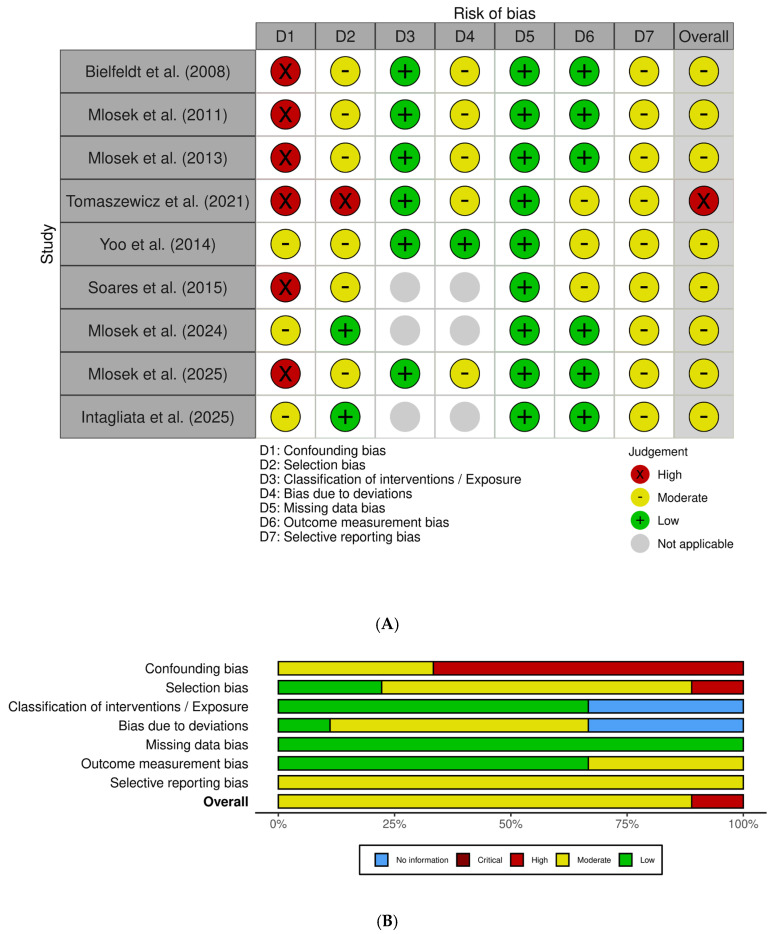
Risk of Bias Assessment—(**A**) Traffic Plot, (**B**) Summary Plot [5,15,17,18,25,26,27,28,29].

As summarized in Figure 2, the overall methodological quality of the included studies was heterogeneous, with all investigations judged as having at least a moderate risk of bias and several presenting a moderate risk of bias in specific domains. The primary sources of bias were related to the insufficient control of confounding variables and limited blinding of outcome assessors.

Six studies [18,25,26,27,28,29] were assessed using the ROBINS-I tool, yielding overall judgments ranging from moderate to high risk of bias. Confounding emerged as the main methodological limitation: none of the studies implemented statistical adjustment for key covariates such as age, BMI, hormonal status, baseline cellulite severity, or lifestyle factors. Selection bias was rated as moderate to serious in most cases, primarily due to the recruitment of highly motivated volunteers from esthetic or dermatologic settings.

Across the included studies, classification of interventions was consistently judged at low risk of bias, supported by the clear and standardized nature of the treatment procedures. Deviations from intended interventions generally presented a moderate risk, largely attributable to limited monitoring of adherence to topical regimens and variable procedural standardization. All studies demonstrated a low risk of bias due to missing data, with high participant retention rates. For outcome measurement, risk was typically moderate because blinding of ultrasonography assessors was rarely implemented or insufficiently reported; nevertheless, the reliance on objective ultrasound parameters partially mitigated this concern. Selective reporting was also judged as moderate, given the absence of preregistered protocols and the heterogeneous depth of reporting across multiple outcomes.

Three additional observational studies [13,15,17] were evaluated using the Newcastle–Ottawa Scale. Soares 2015 [13] showed a moderate risk of bias (5/10), with adequate standardization of imaging procedures but limited control of confounding and a small sample size. Mlosek 2024 [15] also demonstrated moderate risk (6/10), characterized by clear eligibility criteria but the absence of multivariable adjustment, leading to residual confounding. Intagliata 2025 [17] exhibited the lowest risk among the observational studies (7/9), supported by well-defined inclusion criteria, blinded clinical classification, and consistent ultrasound acquisition; however, some residual confounding could not be ruled out.

### 3.4. Results of Individual Studies

#### 3.4.1. Diagnostic Applications of Ultrasound

Five studies investigated the diagnostic applications of ultrasound, encompassing a total of 412 participants (Table 2). Across these investigations, ultrasound consistently demonstrated the ability to quantify structural features associated with cellulite severity, including dermo–subcutaneous interface irregularity, subcutaneous thickness, adipose protrusion depth, dermal echogenicity, and fibrous septa morphology. Early work by Bielfeldt et al. (2008) showed that high-frequency and three-dimensional ultrasound parameters, such as the roughness index, progressively increased with clinical severity, providing initial evidence that ultrasound-derived metrics reflect visually apparent cellulite changes [25]. Subsequent validation and cross-sectional studies confirmed moderate to strong associations between quantitative ultrasound parameters and established clinical grading scales. In particular, subcutaneous thickness, interface length, and adipose protrusion metrics repeatedly correlated with cellulite severity, despite heterogeneity in devices, probe frequencies, and assessment scales [13,15,27]. Importantly, high-frequency ultrasound consistently identified microstructural alterations not readily detectable through surface inspection or photographic assessment alone. More recent work extended the diagnostic role of ultrasound beyond severity grading. Intagliata et al. (2025) demonstrated that multiparametric high-frequency ultrasound can support the subclassification of stage III cellulite, achieving moderate agreement with clinical staging and identifying distinct phenotypes based on fat thickness, septal architecture, edema, and vascularity [17]. Notably, this approach revealed mixed structural patterns not detectable by clinical examination alone.

#### 3.4.2. Ultrasound for Treatment Monitoring

Four studies, including a total of 373 participants, evaluated the usefulness of ultrasound for monitoring treatment response (Table 3). Across interventional investigations, ultrasound consistently detected treatment-related microstructural remodeling of dermal and subcutaneous tissues, with common findings including reductions in subcutaneous thickness, shortening and decreased area of adipose protrusions or hypodermal fascicles, decreased tissue edema, and improvements in echogenicity or elasticity parameters [18,26,28,29]. High-frequency ultrasound proved sensitive to changes induced by diverse therapeutic modalities. In controlled settings, ultrasound-detected structural improvements were observed in active-treatment groups but not in placebo recipients, while in uncontrolled studies, ultrasound findings were generally concordant with improvements in clinical severity scores or circumferential measures. Notably, ultrasound frequently identified early microstructural changes even when clinical improvements were modest, underscoring its value for objective treatment monitoring. More recent comparative studies further demonstrated that high-frequency ultrasound is responsive to differences between treatment modalities, capturing distinct patterns of tissue remodeling across mesotherapy, body wraps, and Endermologie^®^.

### 3.5. Results of Syntheses

Across the nine included studies, substantial methodological and clinical heterogeneity was evident. The investigations differed in ultrasound devices and probe frequencies (ranging from approximately 12 to 35 MHz, with some scanners operating across wider bandwidths), acquisition protocols (including probe pressure, imaging planes, patient positioning, and environmental conditions), and post-processing procedures. Clinical comparators and outcome measures were likewise heterogeneous, encompassing multiple cellulite scoring systems, anthropometric measures, and a broad array of quantitative ultrasound parameters. Given these pronounced differences in interventions, measurement scales, and reporting formats, statistical harmonization and quantitative meta-analysis were deemed inappropriate; therefore, a qualitative synthesis was undertaken. Despite this variability, a coherent pattern emerged indicating that ultrasound consistently identified structural features associated with both cellulite severity and treatment-induced tissue remodeling.

All studies were concordant in demonstrating that ultrasound reliably quantified dermal and subcutaneous morphological characteristics relevant to cellulite. The most frequently assessed parameters included dermal and epidermal thickness, subcutaneous tissue thickness, the length and area of adipose protrusions into the dermis, the morphology and density of fibrous septa, and the presence of edema. Treatment-monitoring studies consistently reported pre–post reductions in dermal and subcutaneous thickness, decreased fat-lobule protrusion, increased dermal echogenicity, and improved tissue elasticity, effects that were detectable even when clinical improvement was modest.

Diagnostic investigations similarly showed consistent associations between ultrasound-derived parameters and clinical cellulite severity. Correlation coefficients ranged from moderate to strong across most cohort studies. One diagnostic study, Intagliata et al. (2025) [17], specifically examined the ability of ultrasound to subclassify stage III cellulite, reporting a satisfactory degree of agreement between ultrasound-based and clinically determined grades, along with acceptable intermethod reliability. The identification of a superficial-fat threshold of approximately 7 mm provided meaningful discrimination between phenotypes 3A and 3B, and the authors additionally described a mixed phenotype that was not detectable through clinical inspection alone.

Across all studies, clinical comparators, including the Nürnberger–Müller scale, photonumeric grading systems, thigh circumference, and standardized photographic assessments, showed moderate alignment with ultrasound findings. Notably, ultrasound often revealed structural abnormalities not captured by clinical grading alone, underscoring its incremental diagnostic value. Elastography further demonstrated the potential to enhance sensitivity by detecting subtle variations in tissue stiffness.

## 4. Discussion

This systematic review synthesizes evidence from nine studies investigating the role of ultrasound in the assessment and monitoring of cellulite. Despite notable methodological heterogeneity, the overall body of evidence consistently supports ultrasound as a reliable and sensitive imaging modality capable of characterizing dermal and subcutaneous structural alterations that mirror clinically relevant features of cellulite. Across observational and interventional designs, ultrasound distinguished between different degrees of severity and quantified treatment-induced tissue remodeling, underscoring its potential as an objective outcome measure in routine clinical practice and research settings [18,30].

From a diagnostic perspective, the available evidence demonstrates that ultrasound can visualize key structural parameters implicated in the pathophysiology of cellulite. Early work by Bielfeldt et al. (2008) [25] showed that three-dimensional ultrasound combined with 20–22 MHz ultrasound could detect dermal–subcutaneous interface irregularities moderately correlated with clinical severity. Subsequent investigations confirmed significant associations between ultrasound-derived metrics and validated grading scales, indicating that quantitative ultrasound measurements reflect clinically meaningful differences in tissue architecture. While some recent work has explored the potential for ultrasound to refine classification of advanced cellulite stages, the broader diagnostic literature—spanning dermatologic imaging and esthetic medicine—consistently highlights the capacity of ultrasound to resolve fine-scale architectural features of the epidermis, dermis, and upper hypodermis with high reproducibility and quantitative precision [31,32].

The diagnostic value of ultrasound is further reflected in the structural patterns consistently observed across interventional studies. A reduction in dermal thickness among women with cellulite, quantifiable with high reliability, is one of the most reproducible findings [15,26]. Subcutaneous depth also varies with severity, reinforcing the ability of ultrasound to capture clinically relevant morphological differences. Of particular importance is the capacity of ultrasound to delineate adipose protrusions extending into the dermis, key contributors to the characteristic dimpled appearance of cellulite. Ultrasound also provides detailed visualization of fibrous septa morphology, a central component of cellulite architecture. Existing evidence shows that septal orientation, thickness, and spatial distribution meaningfully influence severity [33,34], with increased septal thickness and altered configurations frequently documented in affected areas. In addition, ultrasound reliably detects dermal and subcutaneous edema, offering insight into interstitial and inflammatory processes relevant to cellulite pathophysiology [35,36].

A second major theme concerns the responsiveness of ultrasound to therapeutic interventions. Interventional studies consistently reported measurable reductions in dermal and subcutaneous thickness, decreased adipose protrusion height, and improvements in dermal echogenicity following topical, mechanical, or injectable treatments. Mlosek et al. (2013) [18] documented substantial quantitative changes in multiple ultrasound parameters after only 30 days of therapy, while Tomaszewicz (2021) [28] observed significant improvements in skin elasticity and circumferential measures. Similarly, Mlosek and Malinowska (2025) [29] reported ultrasound-detected improvements across mesotherapy, body wrapping, and Endermologie^®^. Several investigations emphasized that ultrasound can identify early microstructural remodeling even when clinical changes are subtle, reinforcing its sensitivity to subclinical tissue dynamics and underscoring the utility of ultrasound in real-time visualization of skin structures and in evaluating parameters such as thickness and vascularity [37], which are essential for accurate monitoring of treatment response.

Notwithstanding convergence in findings, considerable methodological variability was observed across the included studies. Ultrasound probe frequencies ranged from 12 to 35 MHz, with some devices operating across wider bandwidths (20–100 MHz), and imaging approaches varied from conventional two-dimensional B-mode acquisition to three-dimensional reconstruction, strain elastography, and Doppler evaluation. Acquisition protocols were inconsistently standardized, with marked differences in probe pressure, scanning planes, patient positioning, environmental conditions, operator experience, and post-processing procedures. Collectively, these sources of variability may substantially influence the visualization and quantification of dermal and subcutaneous structures, resulting in non-comparable measurements and potentially divergent interpretations of cellulite severity or treatment response across clinical settings. Despite this heterogeneity, concordance between ultrasound-derived parameters and clinical evaluations was generally moderate to strong, with correlation coefficients ranging approximately from r = 0.31 to r = 0.64, and agreement statistics suggesting that ultrasound may enhance classification accuracy beyond visual inspection alone. Nevertheless, the observed variability limits inter-study comparability, precludes robust quantitative synthesis and meta-analytic approaches, and constrains the clinical translation of imaging-derived thresholds, as well as the development of reliable computational and artificial intelligence–based imaging pipelines, which critically depend on standardized acquisition and analytic procedures.

A critical implication of these findings is the need for the development of a standardized ultrasound protocol specifically tailored to esthetic imaging. Future studies should explicitly adopt standardized acquisition and analysis frameworks, including predefined probe frequencies, controlled probe pressure, standardized scanning planes and anatomical landmarks, uniform patient positioning, and clearly defined acquisition and post-processing procedures. Environmental conditions, operator training, and blinding of image assessors should also be systematically addressed.

In addition to technical heterogeneity, an important limitation concerns the insufficient control of key confounding variables, particularly body mass index and age. Both factors influence subcutaneous fat distribution, dermal thickness, tissue stiffness, and microcirculatory features, all of which directly affect ultrasound-derived parameters. As most included studies did not adjust for these covariates, residual confounding cannot be excluded, and some observed associations between ultrasound findings and cellulite severity or treatment response may partially reflect underlying anthropometric or demographic differences rather than cellulite-specific structural changes. Future investigations should therefore incorporate multivariable adjustment or stratified designs to strengthen causal inference and improve the interpretability and comparability of ultrasound-based outcomes.

A further methodological concern emerging from the risk-of-bias assessment relates to the frequent lack of blinding in image acquisition and analysis. In many interventional studies, operators were aware of treatment allocation or follow-up time points, potentially introducing observer-related measurement bias. Although ultrasound provides objective quantitative metrics, image acquisition, region-of-interest selection, and post-processing remain partially operator-dependent. Blinded, independent image analysis, centralized reading, predefined analytic protocols, and a clear separation between image acquisition and interpretation should therefore be prioritized to enhance internal validity and ensure robust outcome assessment.

To our knowledge, this review offers one of the most comprehensive syntheses to date focused exclusively on ultrasound for cellulite assessment. Taken together, the available evidence indicates that, while ultrasound demonstrates strong potential as a non-invasive and objective tool for baseline evaluation and longitudinal monitoring, the current evidence base should be interpreted with caution. Small sample sizes, recruitment predominantly from esthetic settings, and methodological variability restrict generalizability and currently limit the routine clinical implementation of standardized ultrasound-based endpoints. Addressing these challenges through harmonized protocols and rigorous study design represents a necessary step toward broader clinical adoption and future integration of advanced computational and artificial intelligence–based approaches.

The clinical implications of these findings are noteworthy. Ultrasound enables clinicians to move beyond conventional visual grading by providing objective, quantifiable metrics that reflect underlying tissue architecture. The body of evidence synthesized in this review demonstrates that ultrasound introduces a degree of objectivity and morphological resolution not attainable through traditional clinical scales alone. Although tools such as the Nürnberger–Müller scale or photo numeric grading systems remain useful for a global assessment of cutaneous appearance, they are intrinsically influenced by subjective and inter-observer variability. In contrast, ultrasound allows reproducible measurement of structural parameters, including dermal thickness, the depth and area of adipose protrusions, the density and architecture of fibrous septa, and the presence of edema or subtle echostructural alterations. Across included studies, correlations between ultrasound parameters and clinical severity indicate that imaging not only mirrors visible cutaneous differences but also captures microstructural modifications that cannot be assessed through external inspection. This capability is particularly valuable in treatment monitoring, where ultrasound has shown sensitivity in detecting early tissue remodeling even in the absence of overt clinical improvement. From a clinical standpoint, such features allow practitioners to surpass the limitations of purely visual evaluation and to obtain objective metrics that can be contextualized across distinct ultrasonographic phenotypes. Importantly, these insights can be derived without recourse to invasive procedures such as histopathological analysis, which—although potentially informative in selected research scenarios—cannot be justified for routine characterization of cellulite. Ultrasound thus represents a practical, sustainable, and ethically appropriate modality for capturing relevant morphological features in both everyday clinical management and observational research. The ability to identify distinct morphological patterns, including the emerging 3A, 3B, and mixed phenotypes, further expands opportunities for individualized assessment and for deepening understanding of cellulite pathophysiology. It must be acknowledged, however, that the intrinsic phenotypic variability of cellulite and the heterogeneity of available studies do not yet allow a one-to-one correspondence between every ultrastructural parameter and each ecographic subtype. This limitation does not diminish the clinical value of ultrasound; rather, it underscores its strategic role as a tool capable of revealing structural differences that would otherwise remain undetected, providing the preliminary framework upon which more granular and standardized future classifications may be built. In this sense, ultrasound does not replace clinical examination but enhances it and, crucially, lays the scientific foundation for increasingly refined ultrasonographic phenotyping.

As ultrasound technology evolves, with increasing integration of elastography, Doppler imaging, and advanced computational tools, its diagnostic and prognostic capabilities are expected to further expand. Future applications of artificial intelligence and deep learning may support automated image interpretation and longitudinal monitoring, provided that transparency, interpretability, and patient safety remain central to clinical translation.

## 5. Conclusions

This systematic review shows that ultrasound is a valuable imaging modality for characterizing the structural features of cellulite and for monitoring treatment-related tissue remodeling. Across heterogeneous study designs, ultrasound consistently detected changes in dermal and subcutaneous thickness, adipose protrusions, and fibrous-septal architecture corresponding to clinically relevant variations in severity. However, the evidence base remains limited by small sample sizes, methodological heterogeneity, and variable reporting quality. Future research should prioritize standardized ultrasound esthetic imaging protocols, rigorous methodological control, and adequately powered controlled trials incorporating validated clinical and patient-reported outcomes. The establishment of consensus-based ultrasound acquisition and interpretation standards will be essential not only for improving clinical adoption but also for enabling future integration of reproducible and ethically sound AI-based image analysis in cellulite assessment.

## Figures and Tables

**Table 1:** Studies characteristics.

Author	Country	Study Design	Main Aim	Follow-Up	Sample Size	Anatomical Site	Cellulite Severity
Bielfeldt et al. (2008) [25]	Germany	Two cosmetic intervention studies with methodological ultrasound analysis	To standardize non-invasive methods (macrophotography, HF ultrasound) for evaluating efficacy of anti-cellulite products	Study 1: 3 months; Study 2: 4 weeks	Study 1: 36; Study 2: 34	Posterior and lateral thigh	Light to moderate (visual 0–9 scale)
Mlosek et al. (2011) [26]	Poland	Prospective study with treatment vs. placebo groups	To demonstrate applications of classic and high-frequency ultrasonographies in monitoring anti-cellulite therapies	30 days	61 (45 treatment, 16 placebo)	Thighs and buttocks	Cellulite diagnosed clinically; grade not detailed
Mlosek et al. (2013) [18]	Poland	Prospective interventional study with multiple treatment groups and placebo	To assess usefulness of high-frequency ultrasound for monitoring anti-cellulite treatments	30 days (baseline and post-treatment)	84 (66 treatment, 18 placebo)	Posterior thigh	Mean Nürnberger–Müller score 2.89 in treatment group
Yoo et al. (2014) [27]	South Korea	Validation study with two tests (cross-sectional + interventional)	To identify objective parameters highly correlated with visual assessment for evaluating cosmetic anti-cellulite products	6 weeks for Test 2 (measurements at baseline, 2, 4 and 6 weeks)	52 total (Test 1: 20; Test 2: 32, with 28 completing product evaluation)	Thighs (regions with cellulite)	DERMAPRO photonumeric scale grades 1–9 (mild to moderate)
Soares et al. (2015) [13]	Brazil	Cross-sectional diagnostic correlation study	To correlate non-invasive instrumental measures with standardized photographic cellulite severity evaluation	Single visit	26	Buttocks	Grades I–III (Nürnberger–Müller) and photonumeric scale (mild, moderate, severe)
Tomaszewicz et al. (2021) [28]	Poland	Prospective interventional study, pre–post anti-cellulite treatment	To evaluate effectiveness of classic and high-frequency ultrasound in monitoring anti-cellulite therapy	1 month	144	Thigh (widest point)	Grades I–III by Nürnberger–Müller scale (subgroups by severity)
Mlosek and Malinowska (2024) [15]	Poland	Cross-sectional observational correlation study	To determine whether high-frequency ultrasound can aid cellulite assessment and correlate with clinical scores	None (retrospective analysis of single assessment)	114	Posterior thighs	Grades I–III by Nürnberger–Müller scale
Mlosek and Malinowska (2025) [29]	Poland	Prospective comparative effectiveness study with three treatment groups	To assess usefulness of high-frequency ultrasound in evaluating efficacy of three different anti-cellulite treatments	Measurements 14–18 days after completion of therapy series	84 (Group 1: 24; Group 2: 29; Group 3: 31)	Posterior thighs	Grades I–III by Nürnberger–Müller scale at baseline
Intagliata et al. (2025) [17]	Italy	Observational cohort study	To develop and validate an ultrasound-based subclassification of stage III cellulite	None	150	Subgluteal and trochanteric (lateral thigh) regions	Stage III (clinical) classified into ultrasound phenotypes 3A, 3B, Mixed

**Table 2:** Diagnostic applications of ultrasound: summary of included studies.

Study	Ultrasound Method	Frequency/Device	Main Ultrasound Parameters	Key Quantitative Findings	Comparators/Correlations
Bielfeldt et al. (2008) [25]	HFUS + 3D ultrasound	20 MHz; 22 MHz (DUB plus D4W)	Dermis–subcutis roughness (Ra_m_), adipose protrusion depth, borderline length	Ra_m_ correlated with cellulite severity (r = 0.64, R^2^ ≈ 0.41)	Moderate correlation with Smalls 0–9 scale; HFUS captured structural irregularities matching clinical grading
Yoo et al. (2014) [27]	B-mode ultrasound	Not specified	Subcutaneous thickness, dermo-subcutaneous interface length, dermal thickness	Baseline correlations: thickness vs. clinical score r = 0.502; interface length vs. severity r = 0.355; post-treatment correlations: r = 0.31 and r = 0.275	Moderate correlation with photonumeric visual scale; intra-subject active vs. placebo comparison
Soares et al. (2015) [13]	HFUS	20 MHz (DermaScan C)	Dermal thickness, dermal density, interface length, fat herniation	Positive relation between interface length and cellulite severity; dermal density decreased with higher severity	Modest correlation with clinical grading (N–M I–III); strong inter-rater agreement for photographic grading
Mlosek and Malinowska (2024) [15]	HFUS + elastography	18 MHz (Philips Epiq 5); 20–100 MHz (DermaMed)	Subcutaneous thickness, fat protrusion area, epidermis/dermis thickness, elastographic strain ratio	Correlations with severity: subcutaneous thickness r = 0.63; fat protrusions r = 0.64; strain ratio r = 0.51; thigh circumference vs. hypodermis r = 0.48	Among strongest HFUS–clinical correlations reported; good diagnostic consistency
Intagliata et al. (2025) [17]	HFUS + Doppler	20 MHz (Clarius L20 HD3)	Superficial/deep fat thickness, septa density/thickness, edema, vascularity	Diagnostic agreement: 79.2%; Gwet’s AC1 = 0.444; κ = 0.286; α = 0.203; threshold of ~7 mm superficial fat differentiated 3A vs. 3B	Moderate alignment with Nürnberger–Müller scale; HFUS identified “Mixed” phenotype undetected clinically

**Table 3:** Studies employing ultrasound for monitoring treatment response.

Study	Ultrasound Method	Frequency/Device	Main Ultrasound Parameters	Key Quantitative Findings	Comparators/Correlations
Mlosek et al. (2011) [26]	HFUS + classic ultrasound	35 MHz (mScan); 18 MHz (Aplio)	Dermis thickness, subcutaneous thickness, area/length of hypodermal fascicles, edema	Significant reductions in fascicle length and area; decreased hypodermal thickness in treatment group; no change in placebo	HFUS changes aligned with clinical palpation; no numerical correlation provided
Mlosek et al. (2013) [18]	HFUS	35 MHz mechanical	Epidermis and dermis thickness, dermis echogenicity, length/area of subcutaneous bands, edema	Epidermis 0.16 → 0.14 mm; Dermis 1.68 → 1.41 mm; Bands 0.83 → 0.48 mm; Area 0.82 → 0.45 mm^2^; Edema 0.77 → 0.55; Thigh circumference −2.08 cm	Clinical Nürnberger–Müller grade 2.89 → 1.36; ultrasound changes paralleled clinical improvement
Tomaszewicz et al. (2021) [28]	Classic US + HFUS + elastography	12 MHz; HFUS (not specified)	Epidermis/dermis thickness, subcutaneous band length, strain elastography	Elasticity decreased 0.05 units (*p* = 0.032); Thigh circumference −0.76 cm	No control group; ultrasound changes consistent with clinical trends but causality weak
Mlosek and Malinowska (2025) [29]	HFUS + classic US	18 MHz; HF skin scanner (20–100 MHz)	Dermal thickness, echogenicity, subcutaneous thickness, fat protrusion area	Significant reductions in all groups; dermal thickness improved only in body-wrap and Endermologie groups	No explicit correlations; ultrasound strongly responsive to treatment differences

Legend: HFUS: High-Frequency Ultrasound; US: Ultrasound.

## Data Availability

No new data were created or analyzed in this study. All data supporting the findings of this systematic review are derived from published studies cited in the manuscript.

## Supplementary Materials

The following supporting information can be downloaded at: https://www.mdpi.com/article/10.3390/jcm15030943/s1, PRISMA checklist. Reference [24] is cited in the Supplementary Materials.

## Abbreviations

The following abbreviations are used in this manuscript:

AI	Artificial Intelligence
BMI	Body Mass Index
CENTRAL	Cochrane Central Register of Controlled Trials
HFUS	High-Frequency Ultrasound
MRI	Magnetic Resonance Imaging
NOS	Newcastle-Ottawa Scale
PRISMA	Preferred Reporting Items for Systematic Reviews and Meta-Analyses
PROSPERO	International Prospective Register of Systematic Reviews
ROBINS-I	Risk Of Bias In Non-randomized Studies of Interventions
US	Ultrasound
